# Congenital Chylous Ascites in a Neonate with Isolated Aqueductal Stenosis: A Case Report and Literature Review

**DOI:** 10.3390/reports9010086

**Published:** 2026-03-15

**Authors:** Bandar M. Abuageelah, Mona H. Alfaifi, Musaab I. Alnaami, Mubarak M. Alshahrani, Salma M. Jammali, Mohamed F. Hamoda, Mohammed H. Alshehri

**Affiliations:** 1 General Medicine Practice Program, Batterjee Medical College, Obhur 61961, Saudi Arabia; 2 Surgery Department, Saudi German Hospital, Khamis Mushait 62451, Saudi Arabia

**Keywords:** ventriculoperitoneal shunt, lymphatic dysplasia, triglyceride-rich ascites, pediatric neurosurgery

## Abstract

**Background and Clinical Significance**: Ventriculoperitoneal (VP) shunting remains the standard definitive treatment for progressive neonatal obstructive hydrocephalus. Congenital chylous ascites is an uncommon neonatal condition, most often related to developmental lymphatic abnormalities. The concurrence of hydrocephalus requiring VP diversion with congenital chylous ascites is exceptionally rare and may first become apparent during abdominal access for shunt placement. Awareness of this possibility is clinically important because milky peritoneal fluid at shunt surgery can mimic gastrointestinal injury, and persistent postoperative abdominal fluid collections may be misattributed to shunt-related complications. **Case Presentation**: A late-preterm female infant (36 weeks’ gestation; birth weight 2.3 kg) presented with congenital hydrocephalus. Cranial ultrasonography was consistent with isolated aqueductal stenosis. Preoperative abdominal ultrasonography demonstrated mild ascites. On 27 May 2025, a VP shunt was placed for obstructive hydrocephalus. Upon entering the peritoneal cavity, milky-white fluid was encountered, prompting concern for bowel injury; however, careful exploration showed no gastrointestinal perforation. Ascitic fluid analysis revealed markedly elevated triglycerides (2300 mg/dL), confirming chylous ascites. The VP shunt was completed without an intraoperative complication. During follow-up, the infant showed appropriate growth (weight 3.0 kg; length 50 cm), while ascites persisted, and she was referred for multidisciplinary evaluation and management. **Conclusions**: This case highlights an exceptionally rare association of congenital chylous ascites with isolated aqueductal stenosis, identified incidentally during VP shunt insertion. Prompt intraoperative recognition, biochemical confirmation, and coordinated follow-up are essential to distinguish congenital chylous ascites from shunt-related abdominal fluid collections and to guide appropriate multidisciplinary care.

## 1. Introduction and Clinical Significance

Neonatal hydrocephalus is a common and potentially devastating condition that requires timely cerebrospinal fluid (CSF) diversion to prevent progressive ventriculomegaly and neurological injury. In medically fragile or preterm neonates, temporizing CSF drainage (e.g., ventricular reservoir, external ventricular drainage, or ventriculosubgaleal shunt) may be used initially; however, definitive diversion is frequently required when hydrocephalus remains progressive or symptomatic, with ventriculoperitoneal (VP) shunting remaining the most widely used approach in this age group [1,2,3]. Contemporary neonatal cohorts also show that VP shunt placement is often ultimately required when ventricular enlargement persists despite conservative or temporizing strategies [2]. Although endoscopic third ventriculostomy (ETV) is an established alternative for selected obstructive etiologies (including aqueductal stenosis), its effectiveness is age-dependent and generally less reliable in early infancy, supporting VP shunting as the most dependable definitive therapy in many neonates [4].

Chylous ascites is a rare condition characterized by the accumulation of triglyceride-rich lymphatic fluid within the peritoneal cavity. In neonates, primary (congenital) lymphatic disorders, such as intestinal lymphangiectasia or lymphatic dysplasia, predominate, whereas secondary causes (e.g., surgical trauma or postoperative lymphatic disruption) are less frequent but clinically important [5,6,7]. Overall incidence is very low, historically estimated at approximately 1 per 20,000 hospital admissions, and neonatal presentations represent a particularly rare subset [8]. Early recognition is critical because ongoing chyle loss may lead to nutritional, metabolic, and immunologic compromise.

In a targeted literature search of PubMed/MEDLINE and Google Scholar (through January 2026) using terms related to chylous ascites, neonate/infant, hydrocephalus, and ventriculoperitoneal shunt, we found no prior reports describing chylous ascites encountered during VP shunt placement in a neonate with isolated aqueductal stenosis. The coexistence of hydrocephalus requiring VP shunting with chylous ascites is exceptionally rare. A small number of reports describe chylous fluid unexpectedly encountered during VP shunt placement, effectively “unmasking” previously unrecognized chylous ascites at the time of peritoneal entry. Importantly, previously published neonatal or infant cases in this context have typically involved complex congenital anomalies, most notably myelomeningocele and associated defects, rather than isolated aqueductal stenosis [9,10,11]. To our knowledge, VP shunt placement in a neonate with isolated aqueductal stenosis complicated by chylous ascites has not been previously reported.

Accordingly, this report aims to describe a neonate with isolated aqueductal stenosis in whom pre-existing congenital chylous ascites was identified intraoperatively during VP shunt placement, highlighting diagnostic considerations and reinforcing the need to distinguish congenital chylous ascites from postoperative shunt-related abdominal fluid collections.

## 2. Case Presentation

A female infant was born at 36 weeks of gestation with low birth weight and a diagnosis of hydrocephalus. She was delivered in cephalic presentation with clear amniotic fluid. Initial clinical examination revealed equal bilateral air entry on room air, normal first and second heart sounds, a soft and lax abdomen without organomegaly, and an anterior fontanelle at the level with age-appropriate tone, along with a positive symmetrical Moro reflex. The genitalia were normal with a patent anus, the nostrils were bilaterally patent, and the extremities demonstrated good tone and reflexes. A neonatal oral cyst was noted, while the red reflex could not be examined at that time.

**Figure 1:**
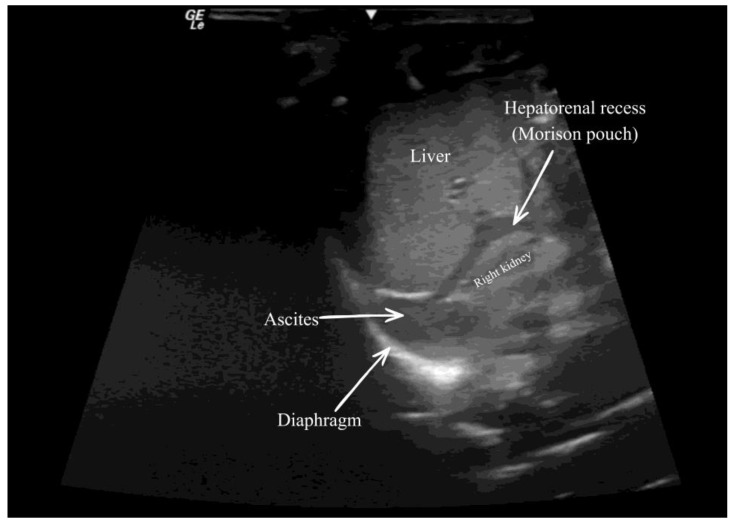
Right upper quadrant (perihepatic) abdominal ultrasound demonstrating free anechoic intraperitoneal fluid (ascites) (arrow) adjacent to the liver and within the hepatorenal recess (Morison pouch) (arrow). The right kidney and diaphragm are labeled for anatomic orientation.

Upon admission, the infant weighed 2.3 kg and was in a moderate state of general health. Cardiovascular examination confirmed normal heart sounds with no murmurs. The respiratory examination demonstrated bilateral adequate air entry on room air, and the abdominal examination again showed no organomegaly. Neurologically, she had a large head with an anterior fontanelle at the level, no seizures, and normal reflexes. Genitalia were consistent with a normal female phenotype. A cranial ultrasound suggested aqueductal stenosis. Additional investigations revealed mild ascites on abdominal ultrasound performed on 23 May (Figure 1), a normal chest radiograph on 25 May, and a repeat cranial ultrasound, which again suggested supporting aqueductal stenosis, and brain MRI was not performed.

**Figure 2:**
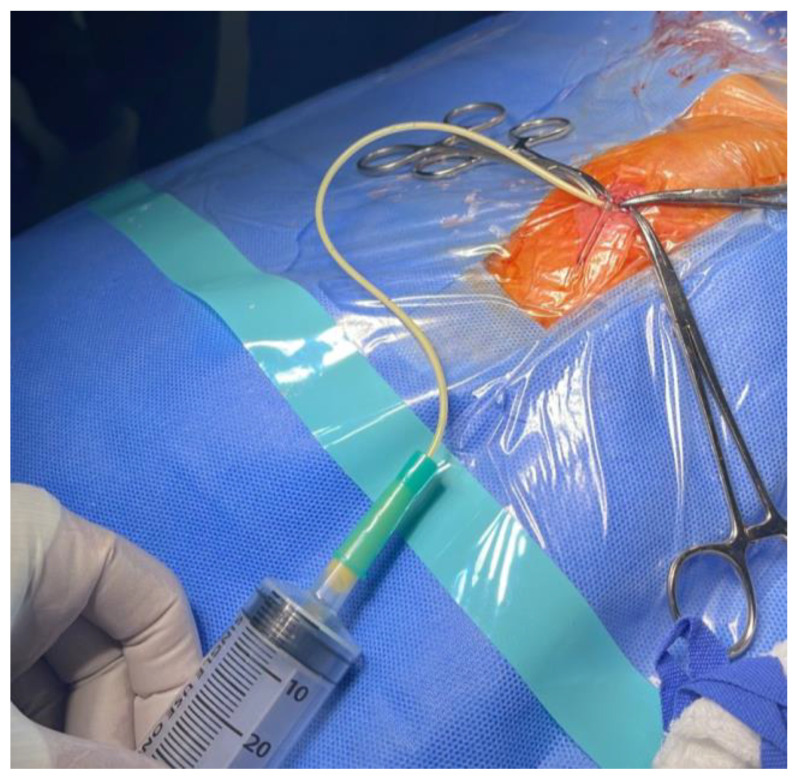
Intraoperative confirmation of chylous ascites during ventriculoperitoneal shunt placement.

On 27 May 2025, with a working diagnosis of obstructive hydrocephalus, the patient underwent a ventriculoperitoneal shunt procedure. Intraoperatively, whitish fluid was observed upon opening the peritoneum, raising concern for possible bowel injury (Figure 2). General surgery was consulted, and a thorough exploration of the stomach and intestines revealed no evidence of injury. Pediatric surgery considered the fluid to represent chylous ascites. A sample was sent for biochemical analysis, which demonstrated markedly elevated triglyceride levels (2300 mg/dL), confirming the suspicion. The remaining ascitic fluid evaluation showed a non-infectious profile, with cell count and differential not suggestive of bacterial peritonitis, protein/albumin within expected limits, and negative microbiological studies (Gram stain/culture). Concurrent serum triglycerides were within the reference range, and chylomicrons were detected according to the available laboratory testing. The ventriculoperitoneal shunt was completed without further complication.

Postoperatively, the infant remained clinically stable and tolerated oral feeds without requiring parenteral nutrition. Therapeutic paracentesis was not required, and albumin supplementation was not needed. Octreotide was not initiated during the index admission, and any additional specialized nutritional interventions were coordinated through the receiving multidisciplinary team. At follow-up, the patient was observed for approximately 22 days after ventriculoperitoneal shunt placement. The infant demonstrated growth (weight 3.0 kg; length 50 cm). On 18 June 2025, sutures were removed, and persistent chylous ascites was noted. She was subsequently referred for continued multidisciplinary follow-up, including pediatric neurology.

## 3. Discussion

This report describes a neonate with obstructive hydrocephalus due to isolated aqueductal stenosis who was found to have triglyceride-rich milky intraperitoneal fluid at the time of VP shunt placement, with biochemical confirmation of chylous ascites. The key clinical nuance is that mild ascites had already been detected preoperatively, supporting a congenital lymphatic etiology rather than a purely postoperative shunt-related abdominal complication. Because VP shunting remains a cornerstone definitive therapy for progressive neonatal hydrocephalus, particularly when temporizing measures are insufficient, recognition of rare coexisting abdominal pathology is essential to avoid unnecessary surgical diversion or misattribution of findings to bowel injury or shunt failure [1,3].

The association between VP shunting and chylous ascites in neonates is exceedingly uncommon, with the available literature limited to isolated reports. Tubbs et al. reported three infants with myelomeningocele who had dark/creamy peritoneal fluid encountered during peritoneal catheter placement shortly after myelomeningocele closure, proposing postoperative lymphatic disruption and/or pressure-related lymphatic rupture as contributing factors [8]. Mavridis et al. similarly described chylous ascites following open myelomeningocele repair in a neonate with hydrocephalus, emphasizing its rarity and noting that VP shunt placement could still be completed safely once visceral injury was excluded [9]. Shumon et al. discussed VP shunting in a neonate with congenital chylous ascites and complex anomalies (including myelomeningocele and imperforate anus), concluding that chylous ascites itself is not necessarily a contraindication to VP shunt placement when appropriate perioperative assessment and follow-up are ensured [10]. In contrast to these reports, our case differs in two clinically important ways: (1) ascites was present before VP shunting, and (2) there were no concomitant spinal dysraphism or major abdominal anomalies, with cranial ultrasound suggesting isolated aqueductal stenosis as the hydrocephalus etiology.

From a pathophysiological standpoint, neonatal chylous ascites is most often related to congenital lymphatic maldevelopment (e.g., lymphatic dysplasia or intestinal lymphangiectasia), resulting in spontaneous chyle leakage into the peritoneal cavity [5]. Contemporary pediatric reviews similarly emphasize that primary lymphatic disorders dominate neonatal etiologies, while secondary causes (surgical trauma, malformations, postoperative lymphatic disruption) are less frequent but clinically relevant [7]. In our patient, the absence of intraoperative bowel injury and the presence of preoperative ascites make a congenital lymphatic leak the most plausible mechanism. VP shunt placement likely unmasked rather than caused the condition; however, it may plausibly have contributed to the persistence or apparent worsening of pre-existing ascites by increasing the intraperitoneal fluid burden through continuous CSF diversion. Available pediatric data indicate that CSF output increases rapidly during the first year of life and may reach several milliliters per hour; in one cohort of infants and children undergoing external ventricular drainage, hourly CSF output ranged from 0.1 to 26.5 mL/h, with a mean of 8.1 mL/h. In a peritoneal cavity with limited absorptive reserve and impaired lymphatic drainage, this additional diverted fluid load may be clinically meaningful [10,11,12]. Accordingly, postoperative ascites should not automatically be interpreted as a primary VP shunt complication without biochemical fluid characterization and careful review of baseline imaging and preoperative abdominal findings.

Diagnostic accuracy hinges on early recognition and biochemical confirmation. Intraoperatively, milky intraperitoneal fluid appropriately triggers concern for visceral injury, enteric contamination, or iatrogenic lymphatic disruption; in such scenarios, prompt surgical evaluation to exclude bowel injury is essential before proceeding. Once chylous ascites is suspected, ascitic triglyceride measurement provides a practical confirmation step; an ascitic triglyceride concentration >200 mg/dL is widely accepted as consistent with chylous ascites, and the markedly elevated level in this case supports the diagnosis [11]. In addition, the preoperative abdominal ultrasound documenting ascites provided crucial contextual evidence that the condition preceded VP shunting. This distinction has practical implications: postoperative abdominal distension after VP shunting may reflect CSF ascites, infection, pseudocyst formation, bowel pathology, or pre-existing non-CSF ascites; therefore, reviewing preoperative imaging and obtaining targeted fluid analysis (triglycerides, cell count with lymphocyte predominance, protein, culture, and-when available-chylomicrons) helps differentiate congenital chylous ascites from shunt-related abdominal complications [5,11]. Where feasible, evaluation for associated congenital anomalies or syndromic lymphatic disease may be warranted, as highlighted in neonatal chylous ascites reviews [5].

Management of neonatal chylous ascites is typically stepwise and conservative at the outset, with escalation reserved for refractory or clinically compromising cases. Rocha’s neonatal review and the pediatric approach summarized by Fawaz et al. emphasize nutritional strategies as first-line therapy: a low–long-chain-fat diet with medium-chain triglyceride (MCT) supplementation (or specialized formulas), high protein intake, and careful monitoring for electrolyte imbalance, hypoalbuminemia, immunoglobulin loss, and poor growth [5,7]. In Kotb et al.’s CARE-compliant pediatric case series, conservative measures and adjunctive therapies (including octreotide) were used, with procedural or shunting approaches considered when medical management failed [6]. Octreotide is commonly described as an adjunct in persistent leaks, and therapeutic paracentesis may be required if respiratory compromise, feeding intolerance, or tense ascites develops [7]. Importantly for neurosurgical decision-making, available case reports indicate that VP shunting can be safely performed in the presence of chylous ascites provided careful intraoperative assessment excludes visceral injury, and postoperative follow-up monitors both shunt function and abdominal status [8,9,10]. Shunt revision to a non-peritoneal terminus should generally be reserved for clearly refractory situations in which the peritoneum cannot accommodate ongoing diversion despite optimal medical management, rather than being an immediate response to discovering chyle.

Clinically, this case reinforces several practical learning points. First, not all neonatal ascites observed during or after VP shunt placement represents a shunt-related complication; preoperative imaging can reveal occult ascites and should be reviewed carefully when unexpected intra-abdominal fluid is encountered. Second, milky intraperitoneal fluid should prompt systematic exclusion of bowel injury, followed by rapid biochemical confirmation of chylous ascites, as triglyceride testing provides a decisive diagnostic discriminator [11]. Third, chylous ascites alone is not necessarily a contraindication to VP shunt placement, consistent with the prior experiences described by Tubbs et al., Mavridis et al., and Shumon et al. [8,9,10]. Finally, coordinated follow-up involving neurosurgery, pediatric surgery, and nutrition is essential to balance durable hydrocephalus control with safe, staged management of the lymphatic leak.

## 4. Conclusions

To our knowledge, this is the first reported association of congenital chylous ascites with isolated aqueductal stenosis in a neonate. The VP shunt procedure revealed an underlying lymphatic disorder, supported by preoperative ascites and confirmatory fluid biochemistry, rather than causing the ascites. Early recognition, triglyceride-based confirmation, and multidisciplinary follow-up are essential, and further case reporting is needed to improve understanding and guide management of this rare neonatal co-presentation.

## Data Availability

The original contributions presented in this study are included in the article. Further inquiries can be directed to the corresponding author.
